# Characterization of Conjunctival Microflora and Antibiotic Sensitivity Patterns in Patients Undergoing Cataract Surgery

**DOI:** 10.3390/microorganisms13020227

**Published:** 2025-01-21

**Authors:** Aldo Vagge, Filippo Lixi, Diego Ponzin, Chiara Del Noce, Davide Camposampiero, Marcello Santocono, Carlo Enrico Traverso, Vincenzo Scorcia, Giuseppe Giannaccare

**Affiliations:** 1 Eye Clinic of Genoa, Policlinico San Martino, Department of Neuroscience, Rehabilitation, Ophthalmology, Genetics, Maternal and Child Health (DiNOGMI), University of Genoa, 16132 Genoa, Italy; aldo.vagge@unige.it (A.V.); chiara.del.noce@medicina.unige.it (C.D.N.); carlo.enrico.traverso@unige.it (C.E.T.); 2 IRCCS Ospedale Policlinico San Martino, 16132 Genoa, Italy; 3 Eye Clinic, Department of Surgical Sciences, University of Cagliari, 09123 Cagliari, Italy; f.lixi1@studenti.unica.it; 4 International Center for Ocular Physiopathology, Veneto Eye Bank Foundation, 30174 Venice, Italy; diego.ponzin@fbov.it (D.P.); davide.camposampiero@fbov.it (D.C.); 5 Ophthalmology Unit (Santocono), Hospital Di Stefano, 95128 Catania, Italy; santocono.m@tin.it; 6 Ophthalmology Unit, Renato Dulbecco Hospital, University Magna Graecia of Catanzaro, 88100 Catanzaro, Italy; vscorcia@unicz.it

**Keywords:** ocular microflora, cataract surgery, antibiotics, antimicrobial resistance

## Abstract

This study aims to characterize the conjunctival flora of patients scheduled for cataract surgery and determine the susceptibility profile of isolated bacteria to several commonly used topical antibiotics. Conjunctival swabs were taken from 44 consecutive patients (25 males, 19 females; mean age of 75.0 ± 12.6 years) who were scheduled for senile cataract surgery at two Italian centers before starting any prophylactic preoperative treatment. Swabs were processed for the detection of the microbial growth and for species identification. Selective culture media were used, and bacteria were identified using the MicroScan Specialty ID Panels (Beckman Coulter^®^, Brea, CA, USA). Antimicrobial susceptibility for the following antibiotics (netilmicin, tobramycin, ofloxacin, oxacillin, levofloxacin, moxifloxacin, chloramphenicol, cefuroxime, and azithromycin) were assessed using the Kirby–Bauer disk diffusion method. Susceptibility for oxacillin was useful to identify methicillin-resistant *Staphylococcus aureus* (MRSA) and methicillin-resistant *Staphylococcus epidermidis* (MRSE). Among the swabs analyzed, 61.4% showed only saprophytic flora, 30.7% showed only potential pathogenic flora, and 8.0% showed mixed flora. *S. epidermidis* (20.5%), *S. intermedius* (18.2%), and *S. aureus* (14.8%) were the most frequent isolates; MRSA and MRSE accounted for 8.0% and 6.8% of isolates. Less frequently (9%), Gram-negative bacteria such as *Pseudomonas fluorescent*, *Serratia marcescens*, *Moraxella lacunata*, *Morganella morgani*, and *Stenotrophomonas maltophila* were detected. All isolated organisms showed an excellent sensitivity to moxifloxacin and chloramphenicol (range 83–100%, range 67–100%, Gram-positive sensitivity for moxifloxacin and chloramphenicol, respectively; 100% Gram-negative sensitivity for both). A significant percentage of the eyes of candidates for surgery presented potential pathogenic flora alone or in association with saprophytic organisms. Staphylococci were the most frequently isolated bacteria. Tobramycin and Ofloxacin, widely used in the ophthalmic field, are confirmed to have a reduced sensitivity in vitro.

## 1. Introduction

The ocular surface is the main eye’s filter of the external environment, serving as a barrier against potentially harmful microorganisms. This defense operates through three primary mechanisms: (i) mechanical, involving the clearance of the ocular surface through blinking and tear production; (ii) chemical, with antimicrobial components in the tears, such as lysozyme, lactoferrin, and defensins; and (iii) immunological, which includes resident immune cells like neutrophils, secretory IgA, and lymphocytes [1].

Moreover, under normal physiological conditions, the ocular surface hosts a huge microbial population, referred to as the “ocular microbiota”. This is composed of both Gram-positive resident microorganisms, such as *streptococci* and *staphylococci*, and Gram-negative bacteria. Most of this microflora consists of non-pathogenic saprophytic organisms that derive nutrients from decomposing organic matter and stimulate immune responses, offering protection against pathogens. Specifically, the ocular saprophytic microflora provides defense through competitive inhibition by occupying space and utilizing nutrients, acting as a natural barrier, and reducing the chances of harmful microbes establishing themselves [2].

Since ocular microflora can be influenced by different parameters such as sex, age, environment, geography, wearing contact lenses, antibiotics use, cosmetics, and diet, the determination of the precise composition of the normal ocular microbiota can be challenging [3]. Disruptions in the ocular microflora, also named dysbiosis, are characterized by the overgrowth of some pathogenic bacteria, which can cause various ocular conditions. Moreover, also, bacteria that are considered non pathological when present in the ocular surface can become pathogenic if introduced into the eye due to the differences in the immunological state between the conjunctiva and the intraocular environment [3,4]. Indeed, bacterial migration during intraocular surgery has been demonstrated in experimental models in which fluoresbrite microspheres (1 µm), mimicking *Staphylococcus* size, spread throughout the anterior chamber and across the intraocular lens (IOL) surfaces during cataract surgery. Despite washing, microspheres persisted on the iris, cornea, and IOL surfaces at the end of surgery, acting as possible vehicle for intraocular infections [4,5]. Therefore, particularly before and after ophthalmic surgery, enhancing ocular hygiene and controlling microbial load on the ocular surface, is crucial to minimize infection risk and to avoid serious postoperative conditions such as endophthalmitis [6,7].

Currently, various strategies for preoperative and postoperative antisepsis are employed to reduce the presence of pathogenic microorganisms on the ocular surface [8]. A 5% povidone iodine (PVI) solution is usually applied immediately before surgery to achieve both periocular and ocular antisepsis; furthermore, an intracameral injection of cefuroxime is administered at the end of cataract surgery [9]. Postoperatively, topical antibiotics are commonly administered for up to 1 week following surgery until the healing of the incisions and should not be tapered for avoiding the risk of selecting antibiotic-resistant organisms [10].

The aims of this study were to characterize the ocular surface microflora and susceptibility profile of the isolated germs in patients scheduled for cataract surgery to identify the appropriate antibiotic regimen and prevent antibiotic resistance.

## 2. Materials and Methods

This study was conducted at the University Hospital of Catanzaro (Catanzaro, Italy), the University Eye Clinic of Genoa, DINOGMI (Genoa, Italy), and the Veneto Eye Bank Foundation (Venice, Italy). Demographic data were collected for each enrolled patient. Exclusion criteria were a history of ocular infection in the studied eye in the last 3 months, the use of glaucoma medications or other types of eye drops for the control of inflammatory conditions (including dry eye), diabetes, and autoimmune diseases. This study received approval by the Ethics Committee of the Calabria Region—Center Area Section on 22 April 2021. All subjects provided written informed consent, and this study was performed in accordance with the Declaration of Helsinki.

Patients undergoing cataract surgery (phacoemulsification with intraocular lens implantation) were enrolled at the University of Eye Clinic of Genoa (Italy) and the University Hospital of Catanzaro (Italy). Conjunctival swabs were performed in all patients scheduled for senile cataract surgery before starting any prophylactic preoperative treatment. As previously described [11], following collection, the conjunctival swab was incubated for 30 min at 32 °C after being combined with Amies transport medium for 15 s. After incubation, the swab had been shaken for further 15 s, and 500 µL of the Amies medium were sterilely injected into an HB&L^®^ culture bottle (Alifax Srl, Padova, Italy). Then, the culture bottles were incubated for a full day in an HB&L^®^ instrument (Alifax Srl, Padova, Italy). Based on the initial nephelometric measurements (CFU/mL) and the matching growth curve produced by the HB&L^®^ instrument, the bacterial load was measured by examining the growth curve. The sample was moved into 1 mL of brain heart infusion broth (BioMérieux, Marcy-l’Etoile, France) if the swab test for microbial growth was positive. It was then incubated for 15 min at 30 °C before being plated on selective media. Non-exigent bacteria or fungi were grown on Sabouraud dextrose agar plates or tryptic soy agar plates, respectively. *Pseudomonas* and *Staphylococcus species* were isolated using cetrimide agar and Baird–Parker agar plates, respectively. Furthermore, exigent bacteria were cultivated on 5% sheep blood agar plates and chocolate agar. Bacteria were incubated for 24–48 h at 37 °C, while fungi were incubated for 5 days at 22–25 °C. The isolated bacteria were identified using MicroScan Specialty ID Panels (Beckman Coulter^®^) and classified as either saprophytic or potentially pathogenic based on references such as *Koneman’s Color Atlas and Textbook of Diagnostic Microbiology* (4th edition) and the *Dizionario di batteriologia umana normale e patologica* (*Dictionary of Normal and Pathological Human Bacteriology*) (Table 1).

In vitro antimicrobial susceptibility for netilmicin, tobramycin, ofloxacin, levofloxacin, moxifloxacin, chloramphenicol, cefuroxime, and azithromycin were tested using the Kirby–Bauer disk diffusion method following the National Committee for Clinical Laboratory Standard Institute protocols [12]. Isolate colonies of each strain were suspended in 2 mL sterile saline, and the suspension was adjusted to 0.5 of the McFarland standard to have approximately 1.5 × 10^8^ bacteria. Bacteria were then transferred with a sterile swab onto Mueller–Hinton agar plates (Oxoid Ltd., Basingstoke, UK) to ensure a confluent lawn of growth. Antibiotic disks (Oxoid Ltd, Basingstoke, UK) for susceptibility assays (netilmicin 30 µg, tobramycin 10 µg, levofloxacin 5 µg, ofloxacin 5 µg, moxifloxacin 5 µg, chloramphenicol 30 µg, cefuroxime 30 µg, azithromycin 15 µg, and oxacillin 5 µg) were placed on the plates using a multidisk dispenser. Susceptibility for oxacillin was useful to identify methicillin-resistant *Staphylococcus aureus* (MRSA) and methicillin-resistant *Staphylococcus epidermidis* (MRSE). Plates were incubated at 37 °C for 24 h, after which the inhibition halos were measured in millimeters. Isolates were categorized as susceptible, intermediate, or resistant using breakpoints set by the European Committee on Antimicrobial Susceptibility Testing (EUCAST) criteria [13]. Only susceptible strains were considered in determining the overall sensitivity.

Statistical analysis was conducted using SPSS for Macintosh software (version 30.0.0.0, SPSS, Inc., Chicago, IL, USA). Means ± standard deviations (SDs) were calculated for numerical continuous variables, while percent distributions were presented for categorical data. Fisher’s exact test was employed to compare categorical variables. A *p* value  < 0.05 was considered statistically significant.

## 3. Results

**Figure 1:**
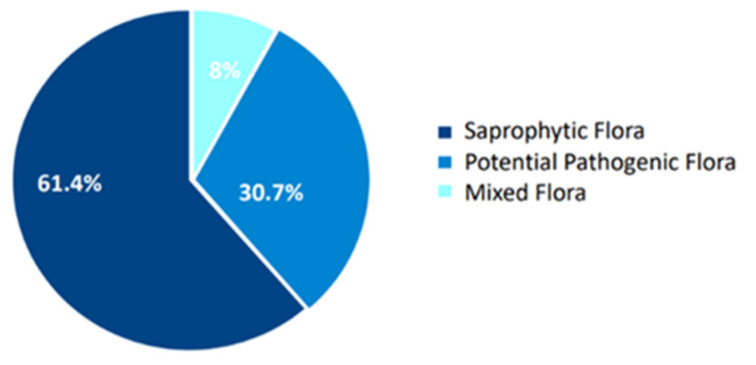
Overall bacterial distribution of swab analysis.

A total of 88 eyes from 44 patients (25 males and 19 females, with a mean age of 75.0  ±  12.6 years) were included in the study. Among swabs analyzed, 54 (61.4% of the total) revealed only saprophytic flora, 27 (30.7%) showed only potentially pathogenic flora, and 7 (8.0%) contained both saprophytic and potentially pathogenic bacteria (mixed flora) (Figure 1).

Of 34 participants with potentially pathogenic flora, 18 (52.9%) were males and 16 (47.1%) were females (*p* = 0.660, Fisher’s exact test). Most of the isolated organisms were Gram-positive (91%). Staphylococcus epidermidis was the most frequently detected strain (18 [20.5%]) followed by Staphylococcus intermedius (16 [18.2%]) and by Staphylococcus aureus (13 [14.8%]); less frequently (9%), Gram-negative bacteria such as Pseudomonas fluorescent (2 [2.3%]), Serratia marcescens (1 [1.1%]), Moraxella lacunata (1 [1.1%]), Morganella morgani (1 [1.1%]), and Stenotrophomonas maltophila (2 [2.3%]) were detected. MRSA and MRSE were detected in 8.0% and 6.8% of conjunctival swabs, respectively. The distribution of the identified bacteria is presented in Table 2.

In vitro susceptibility testing revealed sensitivity/resistance profiles among the isolated bacteria to commonly used antibiotics (Table 3).

Overall, most of the isolated bacteria showed an excellent sensitivity profile to moxifloxacin and chloramphenicol. Staphylococcus epidermidis isolates showed high susceptibility to oxacillin, netilmicin, chloramphenicol, and moxifloxacin, while Staphylococcus aureus isolates were highly susceptible to oxacillin and moxifloxacin. Both species exhibited high resistance to tobramycin, ofloxacin, cefuroxime, and azithromycin. For methicillin-resistant Staphylococcus epidermidis (MRSE) isolates, susceptibility was high only for netilmicin and moxifloxacin, with none showing susceptibility to tobramycin. Methicillin-resistant Staphylococcus aureus (MRSA) isolates were highly susceptible to netilmicin, chloramphenicol, and moxifloxacin. Gram-negative bacteria exhibited high resistance to most antibiotics, except for moxifloxacin and chloramphenicol; both showed 100% sensitivity. Also, netilmicin presented a good sensitivity for Gram, with a range of 50–100%.

## 4. Discussion

The findings of this study highlight the heterogeneity of ocular surface microbiota in patients scheduled for cataract surgery, with a predominance of Gram-positive organisms and variable susceptibility to commonly used antibiotics.

Although cataracts itself does not directly impact the conjunctival flora, factors common to patients with cataracts, such as older age and underlying systemic diseases, can influence the composition of conjunctival bacteria [3]. It is known that individuals often experience modifications in their conjunctival flora throughout their life [14,15]. Wen et al. analyzed ocular microbiota changes in subjects aged from 28 to 84, and reported a greater bacterial diversity in older individuals compared to younger ones, suggesting the influence of difference factors, including hygienic practices, immune status, and interpersonal contacts [14]. Additionally, in a retrospective study of 4432 cataract surgery patients, individuals over 75 years of age showed more conjunctival bacteria compared to younger patients; furthermore, as reported in our cohort, an increased prevalence of Gram-positive bacteria, particularly Staphylococci, was detected [15]. The presence of dry eye in older adults can also affect microbial balance by creating an environment favorable to bacterial growth [3]. Similarly, diabetes mellitus, a systemic disorder common in cataract patients, has been associated with a higher rate of positive bacterial cultures in the conjunctival sac [3]. Hence, patients affected by local and systemic disorders were excluded in our analysis. Moreover, whether a different microbiota composition is present between males and females remains still debated. While Wen et al. suggested that sex may play a role in shaping the conjunctival microbiota [14], Aragona et al. reported that gender has only a secondary influence [3]. In our study, gender did not influence the distribution of the potentially pathogenic flora.

Cataract surgery is the most common surgical procedure performed in all medical specialties, with an estimated 7 million surgeries per year in Europe and 20 million worldwide [16]. One of the most dangerous and sight-threatening complications of cataract surgery is represented by postoperative infections, particularly endophthalmitis, a purulent inflammation of the intraocular fluids comprising the vitreous and the aqueous humor [7].

The etiologic agents of acute postoperative endophthalmitis are typically microorganisms originating from the eyelid margin and tear film that are part of the patient’s own bacterial microflora [17]. In our series, Gram-positive bacteria, which are the main responsible of post-surgical endophthalmitis, were the most frequent isolated bacteria (91%). Indeed, in several studies, coagulase-negative staphylococci (CoNS), such as *Staphylococcus epidermidis*, have been identified as the predominant bacteria on the ocular surface and accounted for approximately 70% of post-cataract surgery endophthalmitis cases, followed by *Staphylococcus aureus*, other Gram-positive bacteria, and Gram-negative microorganisms [17,18,19].

To reduce the microbial load, different strategies are commonly utilized. The European Society of Cataract and Refractive Surgeons (ESCRS) Guidelines consider mandatory the use of PVI applied in the ocular and periocular area prior to surgery [9]. Moreover, the use of intracameral cefuroxime at the end of cataract surgery is supported due to its ability to reduce postoperative cases of endophthalmitis. In recent years, thanks to the routine administration of intracameral cefuroxime, there has been a remarkable decline in the reported rates of postoperative endophthalmitis, dropping from around 0.3–1.2% of the period before its introduction to 0.014–0.08% [9].

However, there is still no global consensus on the optimal approach for infectious prophylaxis in the setting of cataract surgery [20]. In recent decades, prophylaxis with different classes of topical antibiotics has been extensively used [21,22]. This regimen has not shown significant additional benefits beyond intracameral antibiotics, while it has contributed to increasing the number of resistant bacteria [23,24]. Consequently, there has been a progressive shift towards using disinfectants in the preoperative period since they are able to non-selectively reduce microbial load on the ocular surface [25]. Nonetheless, bacteria can survive on the conjunctiva and their detection has been documented in 5.5% of the eyes, even after preoperative washing with PVI [26]. In addition, bacteria are often found at the end of surgery, both in the ocular surface and in the anterior chamber [22,27]. Therefore, the use of postoperative antibiotics remains an effective strategy for reducing infection rates and the likelihood of endophthalmitis [28].

In this analysis, as previously reported [29], Gram-positive strains, including MRSA and MRSE, and the isolated Gram-negative strains exhibited a high in vitro susceptibility to chloramphenicol and moxifloxacin.

Chloramphenicol is an older bacteriostatic antimicrobial agent that was widely used in the past, but which has been partially abandoned due to systemic side effects, such as aplastic anemia. Recently, due to the rising issue of antibiotic resistance, its use as a topical agent has been reconsidered. As an eye drop, chloramphenicol has demonstrated a broad spectrum of activity with a low propensity for inducing bacterial resistance, effective biofilm penetration, and a likely microbiota-preserving action compared to other bactericidal agents [30]. Moxifloxacin is a broad-spectrum antibiotic from the 4th generation of the quinolone class. It targets bacterial DNA by inhibiting replication and repair processes, leading to bacterial death. As an eye drop, moxifloxacin has demonstrated high bioavailability and prolonged retention in ocular tissues, resulting in increased efficacy, improved patient compliance, and a reduced dosing regimen, which has helped preserve the ocular microbiota [31]. Unlike ofloxacin and ciprofloxacin, microbial resistance to moxifloxacin occurs at very minimal rates because it requires the presence of a double mutation on both bacterial enzymes, the DNA gyrase, and topoisomerase [32]. This aspect, together with some characteristics such as tissue concentrations far higher than the MIC, the MSW (mutant selective window), a very low MPC (mutant prevention concentration), and the inhibition of bacterial cell efflux pumps makes moxifloxacin ideal to limit resistance transmission [33].

Netilmicin is a third-generation aminoglycoside that works by binding to the bacterial 30S ribosomal subunit, inhibiting protein synthesis. It is effective against a wide range of pathogens, including resistant strains [29,34]. In the current investigation, bacteria also demonstrated good sensitivity to netilmicin, although some resistant strains were observed, likely due to the widespread use of this antibiotic. This suggests that while netilmicin remains largely effective, its extensive application may have contributed to the emergence of resistance [35].

Conversely, as previously evidenced by other studies [29,30,35], Gram-positive and Gram-negative bacteria, including MRSA and MRSE, presented a reduced sensitivity in vitro to tobramycin and ofloxacin.

Compared to the systemic route, the potential for the development of resistance to topical antibiotic therapy is lower. Yet, the increasing resistance to aminoglycosides and older-generation quinolone has raised significant concerns about the need for a different approach to antibiotic prescription. Accurate diagnosis, targeted therapy, and adherence to guidelines are essential to prevent unnecessary overuse and reduce the risk of resistance. Antibiotics continue to be a key element in infection management, and their role in reducing ocular microorganisms, especially in the postoperative period, should always be highlighted. Nevertheless, continued research into new antimicrobial agents and alternative treatment strategies (e.g., disinfectants) are crucial to effectively counteract antibiotic-resistant bacteria [31,33].

This study has several limitations that should be acknowledged. Firstly, the relatively small sample size limits the generalizability of the findings, and the single time-point sampling (before surgery) may not fully capture the dynamics of ocular flora, which can fluctuate based on environmental and individual factors. Secondly, this study’s setting was limited to two centers in Italy, potentially restricting the applicability of the results to other geographical regions with different bacterial prevalence and resistance patterns. Thirdly, the correlation between in vitro susceptibility findings and clinical outcomes remains unclear, considering that resistance results do not automatically translate into clinical failures.

## 5. Conclusions

The present study confirms the prevalence of Gram-positive bacteria, particularly *Staphylococcus species,* in the ocular surface of patients scheduled for cataract surgery. Approximately one third of isolates were identified as potentially pathogenic, with MRSA and MRSE strains posing a significant risk of infection. These findings underscore the efficacy of moxifloxacin, chloramphenicol, and netilmicin in vitro, suggesting their potential value in perioperative care. On the other hand, the reduced sensitivity to widely used antibiotics such as tobramycin and ofloxacin raises concerns regarding the phenomenon of antibiotic resistance. Given the ongoing rise in resistant bacterial strains, there is a pressing need for judicious antibiotic use and continued exploration of alternative antimicrobial agents such as antiseptic compounds. Future studies involving larger and different populations, with a focus also on postoperative outcomes, should be performed for analyzing prophylactic strategies and developing new therapies to mitigate the risk of postoperative infection and antibiotic resistance in ophthalmic surgery.

## Figures and Tables

**Table 1:** Bacteria classification.

Saprophytic Flora	Potential Pathogenic Flora
*Staphylococcus epidermidis*	*Moraxella lacunata*
*Staphylococcus haemolyticus*	*Morganella morganii*
*Staphylococcus hyicus*	*Pseudomonas fluorescens*
*Staphylococcus intermedius*	*Ralstonia pickettii*
*Staphylococcus lugdunensis*	*Serratia marcescens*
*Staphylococcus simulans*	*Staphylococcus aureus*
*Staphylococcus warneri*	Methicillin-resistant *Staphylococcus aureus* (MRSA)
*Staphylococcus xylosus*	Methicillin-resistant *Staphylococcus epidermidis* (MRSE)
	*Stenotrophomonas maltophilia*

**Table 2:** Distribution of identified bacteria within the entire sample.

Bacteria Isolated	n (%)
*Moraxella lacunata*	1 (1.1%)
*Morganella morganii*	1 (1.1%)
*Pseudomonas fluorescens*	2 (2.3%)
*Ralstonia pickettii*	1 (1.1%)
*Serratia marcescens*	1 (1.1%)
*Staphylococcus aureus*	13 (14.8%)
*Staphylococcus aureus MRSA*	7 (8.0%)
*Staphylococcus epidermidis*	18 (20.5%)
*Staphylococcus epidermidis MRSE*	6 (6.8%)
*Staphylococcus haemolyticus*	5 (5.7%)
*Staphylococcus hyicus*	1 (1.1%)
*Staphylococcus intermedius*	16 (18.2%)
*Staphylococcus lugdunensis*	6 (6.8%)
*Staphylococcus simulans*	3 (3.4%)
*Staphylococcus warneri*	2 (2.3%)
*Staphylococcus xylosus*	9 (10.2%)
*Stenotrophomonas maltophila*	2 (2.3%)
**No growth**	1 (1.1%)
***Saprophytic flora***	54 (61.4%)
***Pathogenic flora***	27 (30.7%)
***Mixed flora***	7 (8%)

**Table 3:** In vitro sensitivity among the isolated bacteria.

In Vitro Sensitivity (%)	OX	NET	TOB	C	OFX	LEV	MXF	CXM	AZM
*Moraxella lacunata* (1)	1 (100)	1 (100)	0	1 (100)	0	1 (100)	1 (100)	0	0
*Morganella morganii* (1)	0	1 (100)	1 (100)	1 (100)	0	1 (100)	1 (100)	1 (100)	0
*Pseudomonas fluorescens* (2)	0	2 (100)	0	0	1 (50)	1 (50)	2 (100)	0	0
*Ralstonia picketti* (1)	0	1 (100)	1 (100)	1 (100)	1 (100)	1 (100)	1 (100)	0	0
*Serratia marcescens* (1)	0	1 (100)	1 (100)	1 (100)	1(100)	1 (100)	1 (100)	0	0
*Stenotroph. malthophilia* (2)	0	1 (50)	2 (100)	2 (100)	2 (100)	2 (100)	2 (100)	0	0
*Staph. aureus* (13)	12 (92.3)	11 (84.6)	3 (23.1)	9 (69.2)	5 (38.5)	7 (53.8)	12 (92.3)	3 (23.1)	1 (7.7)
*Staph. aureus* MRSA (7)	0	6 (85.7)	1 (14.3)	6 (85.7)	3 (42.9)	3 (42.9)	6 (85.7)	1(14.3)	1 (14.3)
*Staph. epidermidis* (16)	16 (100)	15 (93.8)	10 (62.5)	16 (100)	9 (56.3)	11(68.8)	16 (100)	9 (56.3)	1 (6.3)
*Staph. epidermidis* MRSE (6)	0	6 (100)	0	4 (66.6)	1 (16.7)	2 (33.3)	5 (83.3)	2 (33.3)	1 (16.7)
*Staph. haemoliticus* (4)	4 (100)	3 (75)	1 (25)	4 (100)	2 (50)	3 (75)	4 (100)	3 (75)	3 (75)
*Staph. intermedius* (14)	11 (78.6)	10 (71.4)	4 (28.6)	12 (85.7)	5 (35.7)	5 (35.7)	12 (85.7)	6 (42.9)	3 (21.4)
*Staph. lugdunensis* (6)	5 (83.3)	5(83.3)	4 (66.7)	5 (83.3)	1 (16.7)	3 (50)	6 (100)	1 (16.7)	0
*Staph. simulans* (3)	2 (66.7)	2 (66.7)	2 (66.7)	2 (66.7)	0	1 (33.3)	3 (100)	0	0
*Staph. warneri* (2)	1 (50)	1 (50)	1 (50)	2 (100)	0	0	2 (100)	1 (50)	0
*Staph. xylosus* (8)	8 (100)	6 (75)	3 (42.9)	8 (10)	3 (42.9)	4 (50)	7 (87.5)	2 (25)	0

OX: Oxacillin; NET: Netilmicin; TOB: Tobramycin; C: Chloramphenicol; OFX: Ofloxacin; LEV: Levofloxacin; MXF: Moxifloxacin; CXM: cefuroxime; AZM: Azithromycin.

## Data Availability

The original contributions presented in the study are included in the article, further inquiries can be directed to the corresponding author.
